# Changes in habits among mothers during the period of social isolation due to COVID-19 in the municipality of Rio Grande, Rio Grande do Sul, Brazil

**DOI:** 10.1590/1980-549720250055

**Published:** 2025-11-28

**Authors:** Jefferson Sales da Silva, Pâmela Moraes Volz, Rafaela Costa Martins, Francine dos Santos Costa, Cauane Blumenberg, Thais Martins Silva, Romina Buffarini, Lorena Goulart Vieira, Patricia Cota Lima, Zulema Mamani Condori, Caroline Lisset Dominguez Herido, Cielo Amelia Estela-Fernandez, Joaquín Eduardo Chávez Cano, Juraci Almeida Cesar, Rodrigo Meucci, Christian Loret de Mola

**Affiliations:** 1 Universidade Federal de Pelotas, School of Nursing – Pelotas (RS), Brazil.; 2 Universidade Federal do Rio Grande, Health Research and Innovation Group – Rio Grande (RS), Brazil.; 3 Universidade Federal de Pelotas, Human Development and Violence Research Centre – Pelotas (RS), Brazil.; 4 Causale Consultoria – Pelotas (RS), Brazil.; 5 Universidade Federal do Rio Grande, Postgraduate Program in Health Sciences – Rio Grande (RS), Brazil.; 6 Universidade Federal do Rio Grande, Faculty of de Medicine – Rio Grande (RS), Brazil.; 7 Universidad Científica del Sur, Carrera de Medicina Humana, Change Research Working Group – Lima, Perú.; 8 Universidade Federal do Rio Grande, Postgraduate Program in Public Health – Rio Grande (RS), Brazil.

**Keywords:** Women's health, Mothers, Covid-19, Social isolation, Life style, Saúde da mulher, Mães, Covid-19, Isolamento social, Estilo de vida

## Abstract

**Objective:**

To assess the prevalence and determinants of lifestyle habit changes in women during the COVID-19 pandemic.

**Methods:**

This study analyzed data from the 2019 Rio Grande Birth Cohort, a population-based longitudinal study in Rio Grande, Brazil. We used data from the Perinatal Baseline Study (2019), before the COVID-19 pandemic, and the cohort's first follow-up (May–July 2020). We used standardized questionnaires to gather data on maternal sociodemographic characteristics, prenatal care, childbirth, care at birth and changes in habits during the COVID-19 pandemic, including sleep, physical activity, smoking, alcohol consumption, and diet. We used multinomial logistic regression, to calculate relative risk ratios (RR).

**Results:**

In total (n=1,030), 52.5% maintained the same amount of sleep hours, 72.4% did not consume alcoholic beverages, 87.1% were non-smokers, 51.9% reported a decrease in physical activity, and 58.1% reported an increase in food intake. Older mothers, especially those older than 34, had lower risk of sleeping more hours (RR=0.53; 95% confidence interval – 95%CI 0.31–0.89), increasing their physical activity (RR 0.24; 95%CI 0.09–0.62), and were less likely to increase (RR=0.22; 95%CI 0.05–0.99) or decrease (RR=0.14; 95%CI 0.03–0.60) their alcohol consumption.

**Conclusion:**

Social isolation during the pandemic led to changes in maternal habits, especially in physical activity and food intake. Older maternal age emerged as a key predictor, underscoring the need for targeted public health support for mothers during crises.

## RESUMO

**Objetivo:**

Avaliar a prevalência e os determinantes das mudanças nos hábitos de vida de mulheres durante a pandemia de COVID-19.

**Métodos:**

Este estudo analisou dados da Coorte de Nascimentos de Rio Grande de 2019, um estudo longitudinal de base populacional realizado em Rio Grande, Brasil. Foram utilizados dados do Estudo Perinatal Baseline (2019), antes da pandemia de COVID-19, e do primeiro seguimento da coorte (maio–julho de 2020). Questionários padronizados foram empregados para coletar informações sobre características sociodemográficas maternas, cuidados pré-natais, parto, cuidados ao nascimento e mudanças nos hábitos durante a pandemia de COVID-19, incluindo sono, atividade física, tabagismo, consumo de álcool e dieta. Utilizamos regressão logística multinomial para calcular razões de risco relativo (RR).

**Resultados:**

No total (n=1.030), 52,5% mantiveram a mesma quantidade de horas de sono, 72,4% não consumiram bebidas alcoólicas, 87,1% eram não fumantes, 51,9% relataram diminuição na atividade física, e 58,1% relataram aumento na ingestão alimentar. Mães mais velhas, especialmente aquelas com mais de 34 anos, apresentaram menor risco de aumentar as horas de sono (RR=0,53; intervalo de confiança de 95% — IC95% 0,31–0,89), aumentar sua atividade física (RR=0,24; IC95% 0,09–0,62) e eram menos propensas a aumentar (RR=0,22; IC95% 0,05–0,99) ou diminuir (RR=0,14; IC95% 0,03–0,60) o consumo de álcool.

**Conclusão:**

Durante o período de isolamento social, a atividade física e a ingestão alimentar sofreram as mudanças mais significativas entre mães de crianças com até dois anos de idade. Foi observado que a idade avançada foi o mais importante fator preditivo de mudanças nos hábitos das mães.

## INTRODUCTION

Home confinement combined with social distancing were key strategies during the COVID-19 pandemic to avoid viral spread^1^ and eased pressure on the health-care system^2,3^.

However, such actions can have psychological effects on the population. These effects include increased anxiety and depression, especially in countries enforcing the strictest lockdowns^4^, as well as changes in lifestyle. For example, sleep habits may change, and substance use may increase as a coping mechanism^5^.

While mental disorders, especially anxiety and depression, are linked with smoking^6^ and alcohol consumption^7^, current evidence indicates that a slight rise in these addictive behaviors during the COVID-19 pandemic could be attributed to stress, economic hardship, and social isolation^8^. There is no consensus on whether smoking and alcohol consumption increased or decreased during the pandemic^8,9^. Instead, findings indicate a varied response depending on the sociocultural context analyzed, or the consumption patterns of the population^8-10^.

Unhealthy or addictive behaviors are key modifiable risk factors for chronic non-communicable diseases (NCDs)^11^. Individuals with NCDs, along with pregnant women, postpartum women, and the elderly, comprise the risk group for severe forms of COVID-19. Additionally, NCDs are the leading cause of morbidity and mortality globally, and in Brazil they were responsible for more than 54% of deaths in 2019^11^.

In this context of vulnerabilities, the effects of social distancing on mothers of young children were particularly complex and often overlooked. These women faced not only the inherent stress of isolation but also an excessive burden of household and childcare duties, the loss of social support for child-rearing, disrupted sleeping and dietary patterns, and numerous challenges at work, whether to maintain their jobs or perform them safely^12^. This combination of factors helps explain the sharp rise in mental health issues within this group^13^, which likely had a notable impact on their daily habits.

While a significant body of research has investigated the general health impacts of the pandemic, few studies have assessed these changes specifically in mothers within the Brazilian context. Therefore, the aim of this study was to assess the prevalence and factors linked to changes in habits related to smoking, food intake, alcohol consumption, sleep time, and physical activity during the initial months of the COVID-19 pandemic in women whose children were born in 2019 in the municipality of Rio Grande, state of Rio Grande do Sul, Brazil.

## METHODS

This study used data from the 2019 Rio Grande birth cohort, a population-based longitudinal study in Rio Grande, Southern Brazil. We analyzed data from the Perinatal Baseline Study and Wave I of the WebCOVID study, both described later.

The 2019 Perinatal Study documented all births occurring between January 1 and December 31, 2019, at two hospitals in Rio Grande, RS, Brazil: Santa Casa de Misericórdia de Rio Grande (SCMRG) and the University Hospital of the Federal University of Rio Grande (HU-FURG). These two facilities account for more than 99% of all births in the city. Within 48 hours of delivery, mothers were invited to complete a standardized questionnaire designed to evaluate sociodemographic characteristics of the mother and family, prenatal care, and childbirth care. The survey included responses from 2,314 mothers, of which 2,052 met the inclusion criteria for the 2019 Birth Cohort: residing in the city's urban area, having delivered a liveborn from a single fetus weighing ≥ 500 grams or with a gestational age of at least 20 weeks.

The WebCOVID study, which originated from the 2019 Birth Cohort, includes a series of follow-ups conducted from 2020 to 2023. Specifically for this study, we used data from the first follow-up, which occurred from May 11 to July 20, 2020. Of the 2,051 mothers eligible for this follow-up, 1,030 participated. Losses to follow-up (n=1,021) included mothers who could not be located or who refused to participate. To locate the mothers and invite them to participate, the research team made at least three contact attempts at different times via web or telephone and also utilized social networks. Of the 1,021 mothers who were not included in the analysis, approximately 90% (n≈919) were mothers we were unable to contact, and the remaining 10% (n≈102) explicitly rejected participation (Supplementary Table 1).

The study employed a standardized online questionnaire, created using Research Electronic Data Capture (REDCap^®^), with an average completion time of ten minutes. Trained interviewers invited mothers to participate via phone calls, WhatsApp messages, and social media platforms such as Facebook and Instagram. The research team made at least three contact attempts at different times for each eligible participant. The interviewers remotely sent the questionnaire link for completion and were also trained to administer the survey by phone if necessary.

Further details on this and other follow-ups can be found in Martins et al.^14^.

### Variables

We evaluated the main outcomes, which included changes in habits related to sleeping hours, physical activity, smoking, alcohol consumption, and food intake through the question: "Since the beginning of the pandemic, have you changed the number of hours you sleep?" (yes, increased; yes, decreased; no, remained the same); "…has the amount of physical activity/exercise changed?" (yes, it has increased; yes, it has decreased; no, it remains the same); "…have you changed how much you smoke?" (no smoking; yes, increased; yes, decreased; no, remained the same); "…have you changed the amount of alcoholic beverages consumed?" (no drinking; yes, increased; yes, decreased; no, remained the same); "…have you changed how much you eat?" (yes, increased; yes, decreased; no, remained the same).

We evaluated changes in habits related to sleeping hours, physical activity, smoking, alcohol consumption, and food intake using a standardized online questionnaire. For each behavior, mothers were asked a simple question, such as: "Have you changed the number of hours you sleep?" The response options were categorical: "Yes, increased," "Yes, decreased," or "No, this has remained the same." This approach was designed to capture the direction of change in behaviors during the social isolation period rather than their magnitude. It is important to note that our assessment of physical activity was a general measure, and alcohol consumption was assessed categorically without distinguishing between sporadic or abusive use.

We used socioeconomic and demographic characteristics from the baseline Perinatal Study as independent variables: we categorized maternal age into four groups (13–19; 20–24; 25–34; 35 or more) based on complete years. We grouped maternal schooling by complete years of formal education (0–8; 9–11; 12 or more). We classified maternal skin color as white, brown, or black. We measured family income in Brazilian *reais* by summing the monthly salaries of all family members and categorizing them into four groups (R$ 0–1,000; 1,001–2,000; 2,001–3,000; 3,001 or more).

### Statistical analysis

We described both independent and outcome variables using absolute and relative frequencies. To assess potential bias due to loss to follow-up in the cohort, we compared the characteristics of the analytical sample with those of the individuals not included in the analysis. We performed all comparisons using Fisher's exact test. We described the prevalence (%) of changing habits according to the categories of independent variables. To assess the association between socioeconomic and demographic characteristics and each of the outcomes, we performed multinomial logistic regression models, generating the relative risk (RR) as a measure of effect. This measure assesses how many times lower or higher the risk ratio of the categories "increased" and "decreased" was in comparison with the category "same" (no changes during the COVID-19 pandemic — chosen as the reference outcome category).

To address possible selection bias in our cohort, we applied propensity score analysis and inverse probability weighting (IPW) to generate an index summarizing the likelihood of being followed, based on baseline sociodemographic characteristics. This variable was included in our final analyses to calculate p-values and 95% confidence intervals reported in our publications. All multivariable analyses were weighted using the results from this IPW procedure. The weights ensured that each participant contributed appropriately to the analysis relative to their probability of being followed. In our sample, it was clear that having certain characteristics made it more or less likely for an individual to be included; thus, individuals with characteristics that made them less likely to be included in the follow-up were assigned a higher weight than those more likely to participate. This approach helps to correct for potential selection bias and provides more accurate and generalizable estimates.

To select variables for our models, we used a theoretical framework, assuming that maternal age, schooling, skin color, and family income were precursors to both the main exposures and the outcomes. We did not use a statistical cutoff for variable inclusion. We assessed model adequacy by using a goodness-of-fit test to check for potential overfitting.

We conducted all the analyses using Stata version 18.0 statistical software (StataCorp LLC, College Station, TX, USA) and considered a 5% significance level for the results (p-value).

The ethics committee of the Universidade Federal do Rio Grande approved the research under protocol no. 15724819.6.0000.5324.

### Data availability statement

The entire dataset supporting the findings of this study is available upon request to the corresponding author.

## RESULTS

In total, we evaluated 1,030 mothers. We observed statistically significant differences regarding all socioeconomic and demographic characteristics when comparing the participants included with those not included in the analysis (Supplementary Table 1). The analyzed sample comprises 50.2% of the total population, primarily consisting of women aged 25 or over (64.8%). A significant proportion of women in the sample have white skin color (79.9%) and higher family income (30.6%) (Table 1).

**Table 1:** Socioeconomic and demographic characteristics of included participants (n=1,030) from Rio Grande, Brazil.

Variables		n (%)
Maternal age (years)		
	13–19	107 (10,4)
	20–24	256 (24,9)
	25–34	484 (47,0)
	35 or more	183 (17,8)
Maternal schooling (years)		
	0–8	220 (21,4)
	9–11	501 (48,6)
	12 or more	309 (30,0)
Maternal skin color		
	White	823 (79,9)
	Brown	144 (14,0)
	Black	63 (6,1)
Family income (R$)		
	0–1,000	115 (11,4)
	1,001–2,000	329 (32,5)
	2,001–3,000	256 (25,4)
	3,001+	310 (30,7)

In the study, 52.5% of mothers reported maintaining their usual number of hours of sleep. A significant majority indicated that they did not smoke (87.1%) or consume alcohol (72.4%), thus maintaining previous behaviors (Table 2). Nearly 52% of participants reported a reduction in physical activity, whereas approximately 60% noted an increase in food consumption.

**Table 2:** Changes in habits and behaviors during the COVID-19 pandemic, Rio Grande 2019 Cohort, Brazil (n=1030).

Change in habits		n (%)
Sleeping hours		
	Same	541 (52,5)
	Increased	167 (16,2)
	Decreased	322 (31,3)
Physical activity practice		
	Same	419 (40,7)
	Increased	76 (7,4)
	Decreased	535 (51,9)
Smoking		
	I don't smoke	897 (87,1)
	Yes, it has increased	73 (7,1)
	Yes, it has decreased	14 (1,4)
	No, it's still the same	46 (4,5)
Alcohol consumption		
	I don't drink	746 (72,4)
	Yes, it has increased	56 (5,4)
	Yes, it has decreased	56 (5,4)
	No, it's still the same	172 (16,7)
Food intake		
	Same	327 (31,7)
	Increased	598 (58,1)
	Decreased	105 (10,2)

Supplementary Figures 1 to 5 show the association between changes in habits and behaviors and the socioeconomic and demographic characteristics of the sample. Younger women (aged 13 to 24) and women earning between R$ 1,001.00 and R$ 3,000.00 report a higher prevalence of increased sleep hours (21.0, 21.8, and 17.8% respectively). Additionally, mothers aged 25 to 34, those with 12 or more years of schooling, and those with an income of over R$ 3,000.00 show a greater reduction in physical activity (53.9, 59.5, and 57.7% respectively) (Supplementary Figure 2).

Women with up to eight years of schooling (11.3%), a family income between R$0 and R$1,000 (12.1%), and those with brown skin color (15.2%) increasingly smoked more cigarettes (Supplementary Figure 3). Younger women aged 13 to 24 (7.4%) and those with up to eight years of schooling (7.2%) more frequently reported a decrease in alcohol consumption (Supplementary Figure 4). Although the changes in the amount of food eaten according to sociodemographic and economic characteristics did not show statistically significant differences, the variable still exhibited one of the greatest changes in the direction of increased food consumption (Supplementary Figure 5).

Multivariable analysis revealed that, compared to women aged 13–24, mothers aged 25–34 had a 37% lower risk (RR=0.63; 95% confidence interval — 95%CI 0.44–0.93) of increasing sleep hours, and those aged 35 or older had a 47% lower risk (RR=0.53; 95%CI 0.31–0.89) (Table 3).

**Table 3:** Association between socioeconomic and demographic characteristics and changes in lifestyle habits during the COVID-19 pandemic. Rio Grande 2019 Cohort.

		Sleeping hours		Physical activity practice		Smoking	
		Increased RR (95%CI)	Decreased RR (95%CI)	Increased RR (95%CI)	Decreased RR (95%CI)	Increased RR (95%CI)	Decreased RR (95%CI)
Maternal age (years)							
	13–24	1	1	1	1	1	1
	25–34	0.63 (0.44–0.93)	1.13 (0.83–1.54)	0.71 (0.43–1.19)	1.14 (0.85–1.52)	0.97 (0.44–2.13)	0.58 (0.17–2.04)
	35 or more	0.53 (0.31–0.89)	0.95 (0.63–1.42)	0.24 (0.09–0.62)	0.91 (0.63–1.32)	0.61 (0.15–2.41)	0.49 (0.05–4.94)
Maternal schooling (years)							
	0–8	1	1	1	1	1	1
	9–11	0.64 (0.42–0.99)	0.79 (0.55–1.14)	0.98 (0.52–1.87)	1.35 (0.97–1.87)	1.03 (0.47–2.92)	0.79 (0.23–2.78)
	12 or more	0.67 (0.42–1.08)	0.88 (0.60–1.30)	2.06 (1.05–4.01)	2.19 (1.51–3.16)	1.36 (0.39–4.69)	0.57 (0.05–5.88)
Maternal skin color							
	White	1	1	1	1	1	1
	Brown	1.51 (0.95–2.40)	0.87 (0.57–1.31)	2.13 (1.16–3.90)	1.05 (0.72–1.53)	3.48 (1.21–10.06)	2.28 (0.46–11.20)
	Black	2.02 (1.06–3.83)	1.04 (0.56–1.90)	1 (0.37–2.67)	0.63 (0.37–1.07)	0.59 (0.13–2.82)	0.95 (0.10–9.47)
Family income (R$)							
	0–1,000	1	1	1	1	1	1
	1,001–2,000	1.60 (0.86–2.97)	0.80 (0.50–1.28)	2.18 (0.86–5.54)	1.37 (0.89–2.13)	1.56 (0.59–4.13)	4.94 (0.54–45.58)
	2,001–3,000	1.28 (0.67–2.43)	0.87 (0.53–1.41)	1.11 (0.41–3.01)	0.97 (0.62–1.52)	3.14 (0.95–10.30)	8.00 (0.73–88.23)
	3,001+	0.66 (0.34–1.29)	0.82 (0.51–1.30)	2.04 (0.79–5.28)	1.67 (1.07–2.60)	0.34 (0.09–1.38)	2.40 (0.19–30.52)

Mothers with 12 or more years of education had a higher risk (RR=2.19; 95%CI 1.51–3.16) of changing their physical activity levels compared to those with eight or fewer years of education (RR=2.06; 95%CI 1.05–4.01).

Compared to mothers self-reported as white, mothers self-reporting as brown significantly increased their tobacco consumption (RR=3.48; 95%CI 1.21–10.06) and those self-reported as black increased their sleep hours (RR=2.02; 95%CI 1.06–3.83) (Table 3).

Age was the only maternal characteristic linked to changes in alcohol consumption. Mothers aged 35 or older had a 78% lower risk (RR=0.22; 95%CI 0.05–0.99) of increasing their alcohol intake during the pandemic and an 86% lower risk (RR=0.14; 95%CI 0.03–0.60) of decreasing their consumption compared to younger mothers, indicating a relative stability in their alcohol consumption. No significant associations were found between maternal characteristics and changes in food intake (Table 4).

**Table 4:** Association between socioeconomic and demographic characteristics with changes in alcohol consumption and food intake.

		Alcohol consumption			Food intake		
		Increased RR (95%CI)	Decreased RR (95%CI)	Increased RR (95%CI)	Decreased RR (95%CI)
Maternal age (years)					
	13–24	1	1	1	1
	25–34	1.91 (0.98–3.71)	0.88 (0.47–1.65)	0.77 (0.57–1.04)	0.89 (0.55–1.44)
	35 or more	0.22 (0.05–0.99)	0.14 (0.03–0.60)	0.71 (0.48–1.04)	0.51 (0.25–1.02)
Maternal schooling (years)					
	0–8	1	1	1	1
	9–11	0.61 (0.25–1.45)	0.47 (0.22–1.01)	1.08 (0.76–1.53)	0.75 (0.44–1.28)
	12 or more	1.25 (0.53–2.97)	0.45 (0.19–1.05)	0.94 (0.64–1.38)	0.66 (0.36–1.19)
Maternal skin color					
	White	1	1	1	1
	Brown	0.95 (0.43–2.10)	0.68 (0.27–1.62)	1.02 (0.69–1.51)	0.89 (0.46–1.73)
	Black	0.54 (0.11–2.52)	1.34 (0.44–4.08)	0.99 (0.55–1.77)	1.66 (0.74–3.71)
Family income (R$)					
	0–1,000	1	1	1	1
	1,001–2,000	0.84 (0.28–2.49)	0.73 (0.27–1.98)	1.20 (0.75–1.92)	1.23 (0.58–2.61)
	2,001–3,000	0.74 (0.23–2.34)	0.51 (0.17–1.52)	0.94 (0.58–1.52)	1.12 (0.52– 2.43)
	3,001+	0.70 (0.24–2.08)	0.36 (0.13–1.04)	0.94 (0.59–1.51)	0.75 (0.34–1.63)

RR: relative risk.

## DISCUSSION

Our research indicated that sociodemographic and economic factors affected maternal behaviors during the COVID-19 pandemic. Notably, physical activity and dietary habits were the most significantly altered behaviors, highlighting the influence of these factors on mothers during social isolation. However, none of the variables studied could predict changes in dietary habits, and age was a predictor of changes in physical activity.

The study indicated that over half of the women interviewed maintained their sleep hours during social isolation, aligning with findings by Holzinger et al.^15^ about the general population. Sleep is regarded as an essential function for living beings and one of the fundamental pillars of health, affecting both physical and mental well-being^16^.

Women over 25 years old experienced a reduced risk of increasing sleep hours. An Ibero-American study also observed an increase in sleep hours within the general population, particularly among individuals under the age of 30^2^. This outcome may be attributed to differing responsibilities and priorities across various age groups. Furthermore, among women with higher income, financial stability and the option to work remotely may have contributed to this result. The study by Lima et al.^17^ indicated that sleep quality issues during the COVID-19 pandemic were more prevalent among women with a monthly *per capita* income of less than one minimum wage, in addition to an increase in household chores during the pandemic; however, this was not reflected in our study.

As previously noted, there was a reduction in physical activity among the mothers interviewed. A study conducted in Rio de Janeiro, Brazil, during the COVID-19 pandemic found that individuals who had a child during this period were 35% less likely to engage in physical activity compared to those without children^18^. Physical activity provides both physical and psychological benefits and is linked to a lower risk of noncommunicable diseases and improved outcomes during pregnancy and childbirth. Consequently, regular physical activity, including during the postpartum period, is positively correlated with enhanced health and well-being for both mothers and their children^19^.

Consistent with Malta et al., a decline in physical activity was noted among higher-educated groups, due to closures of public spaces and increased maternal responsibilities during the pandemic^20^. Higher educated individuals also tend to have more sedentary jobs and are more aware of COVID-19 risks, contributing to reduced activity levels among mothers^18^. Conversely, an increase in physical activity was also predicted by a higher education level, indicating that higher educated women were more likely to change their physical activity habits. It is likely that higher educated women who performed more activity before the pandemic drastically reduced their activity during the social isolation period, while those who were previously more sedentary used this time to increase their physical activity to stay active, as the extended time at home could feel confining.

In the present study, 72.4% of mothers did not consume alcohol. This result may be explained by the closure of leisure establishments and reduced social interactions during the pandemic, which limited alcohol availability. Additionally, among women who consumed alcohol during the pandemic, mothers aged 35 years or older had a lower relative risk of increased consumption compared to younger mothers. This trend is consistent with findings that older mothers often have more cautious attitudes toward alcohol consumption, likely due to greater awareness of risks to their younger children's health and well-being.

In contrast to alcohol consumption, food intake significantly increased during the pandemic. A study found that 46% of the population had increased their intake during the lockdown compared to the period before it began^21^. Women were found to be less likely than men to maintain a stable food intake; they reduced the frequency of consuming vegetables and fruits while increasing the intake of snacks and bakery products throughout the pandemic^2^. This increased consumption of low-nutrient foods may raise the risk of overweight or obesity in women, potentially leading to complications and health conditions, including maternal-fetal issues^22^.

Our study has certain limitations, including a loss to follow-up of nearly 50%, which is frequently observed in online and remote research environments. Additionally, the limited socioeconomic diversity resulting from these losses within our sample may have resulted in an underestimation of changes in habits among mothers with lower income and education levels, who, as noted in previous studies, experienced greater effects during the COVID-19 pandemic. We sought to reduce the potential for selection bias by applying weights derived from IPW analysis, as detailed in the statistical analysis section.

Another limitation of our study is the use of a survey design that captures only the direction of change in habits (increased/decreased) rather than the magnitude of these changes. This approach, while providing a general overview, does not allow us to determine the quantitative impact of the pandemic on specific behaviors or whether the final estimates were high or low.

Furthermore, our assessment of both physical activity and alcohol consumption relied on general categorical questions. Regarding physical activity, this was a deliberate choice to capture overall changes in activity during the specific and restrictive context of quarantine, when all forms of activity were likely to be affected. Our assessment of alcohol consumption did not distinguish between sporadic and abusive use. We also acknowledge that our high loss to follow-up, a common challenge in online research, may affect the representativeness of our findings.

Despite these limitations, our study offers several strengths. It is the first to evaluate the prevalence and factors associated with changes in habits during the initial months of the COVID-19 pandemic among mothers of infants, focusing on smoking, food intake, alcohol consumption, sleep time, and physical activity. Additionally, it is one of the few online birth follow-ups conducted in Brazil and Latin America, and the only one carried out during the COVID-19 pandemic. Therefore, it provides valuable evidence on changes in maternal practices in Brazil during this period.

This study provides key insights into how the COVID-19 pandemic and subsequent social isolation measures impacted maternal health behaviors. We found that socioeconomic and demographic factors significantly influenced specific changes in maternal habits related to sleep, physical activity, and smoking. For instance, increased sleep hours were associated with higher maternal age and family income, while changes in physical activity were linked to both income and maternal education. These findings underscore significant health behavior disparities during a public health crisis and highlight the need for targeted strategies to support maternal well-being in similar future events. The study's results can be used to inform public health strategies that address these specific vulnerabilities.
