# Supplementary material for: Changes in habits among mothers during the period of social isolation due to COVID-19 in the municipality of Rio Grande, Rio Grande do Sul, Brazil
Source: Rev Bras Epidemiol. 2025 Nov 28;28:e250055. doi: 10.1590/1980-549720250055 (PMC12667504; doi:10.1590/1980-549720250055)

Supplementary Table 1: comparison of the socioeconomic and demographic characteristics of the sample included and not included in the analyses.

	Sample not included in the analysis (n=1,021)	Analytical sample analyzed (n=1,030)	*p value*
	**n (%)**	**n (%)**	
**Maternal age (years)**			< 0.001
13-19	148 (14.5)	107 (10.4)	
20-24	300 (29.4)	256 (24.9)	
25-34	426 (41.7)	484 (47.0)	
35 or more	147 (14.4)	183 (17.8)	
**Maternal schooling (years)**			< 0.001
0-8	387 (37.9)	220 (21.4)	
9-11	476 (46.6)	501 (48.6)	
12 or more	158 (15.5)	309 (30.0)	
**Maternal skin color**			< 0.001
White	753 (73.8)	823 (79.9)	
Brown	164 (16.1)	144 (14.0)	
Black	104 (10.2)	63 (6.1)	
**Family income (R$)**			< 0.001
0-1000	196 (19.8)	115 (11.4)	
1001-2000	371 (37.3)	329 (32.6)	
2001-3000	234 (23.6)	256 (25.4)	
3001+	191 (19.3)	310 (30.6)	

Supplementary Figure 1. Association between the change in sleeping hours and family income, age, education, and maternal skin color. Data collected from women in Rio Grande, Brazil, who gave birth in 2019, with follow-up from May 11 to July 20, 2020.

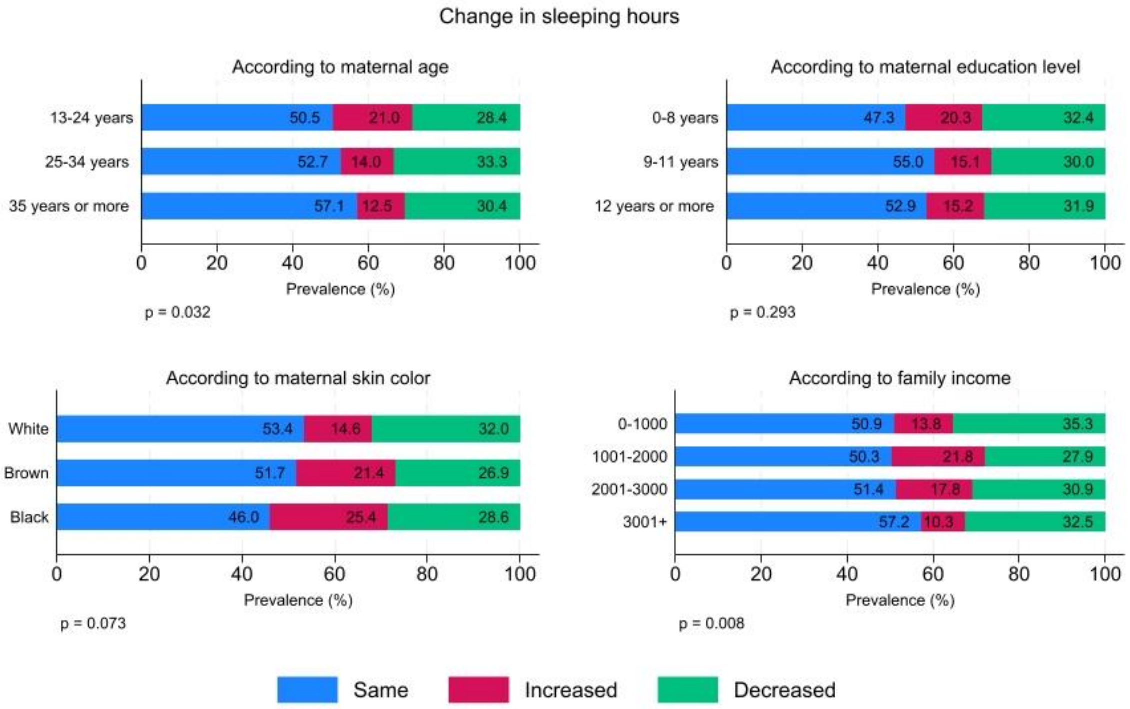

Supplementary Figure 2. Association between changes in physical activity practices and household income, age, education, and maternal skin color. Data collected from women in Rio Grande, Brazil, who gave birth in 2019, with follow-up from May 11 to July 20, 2020.

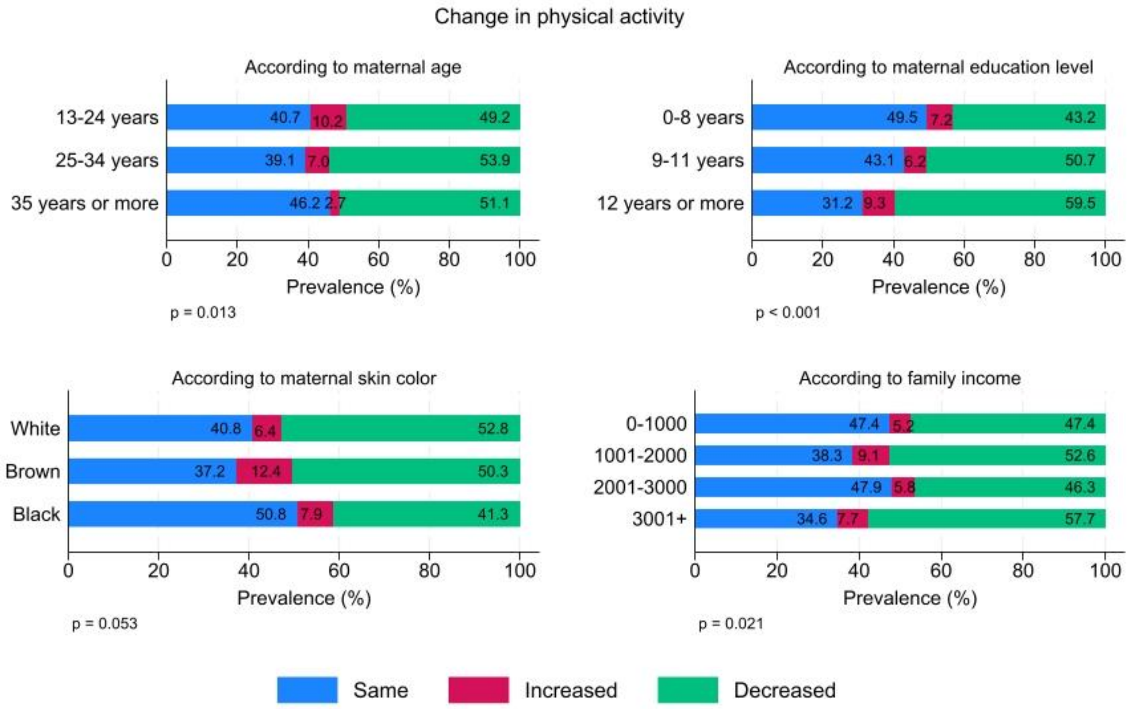

Supplementary Figure 3. Association between the change in smoking habits and family income, age, education, and maternal skin color. Data collected from women in Rio Grande, Brazil, who gave birth in 2019, with follow-up from May 11 to July 20, 2020.

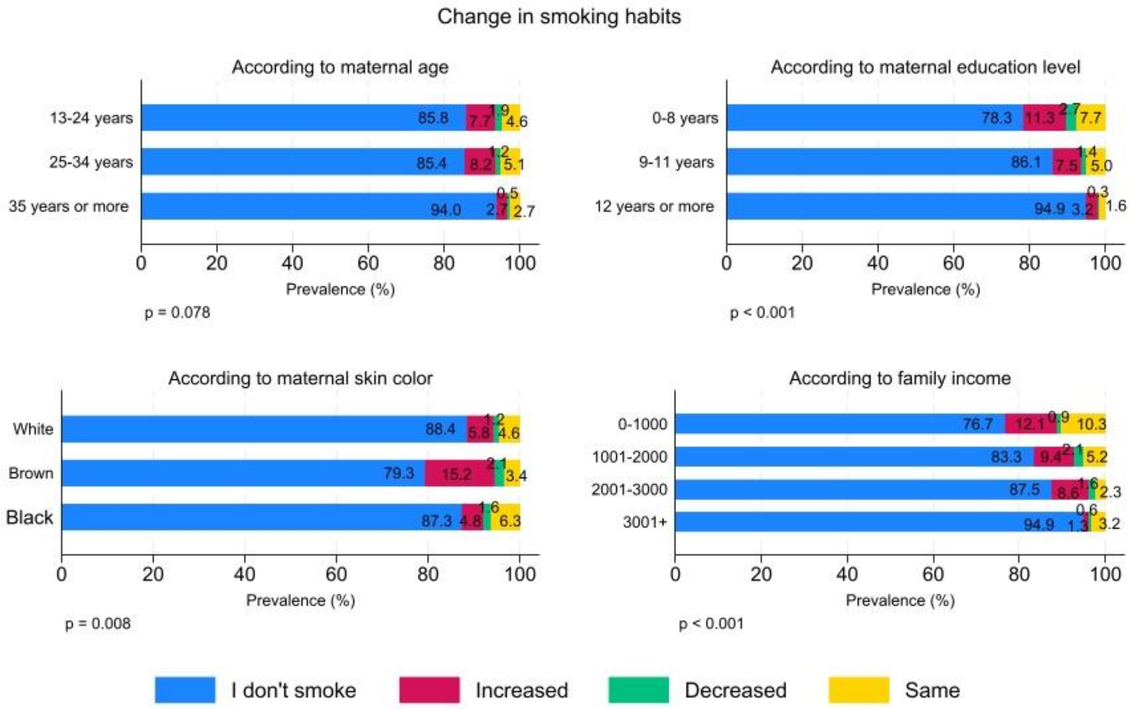

Supplementary Figure 4. Association between the change in alcohol consumption and family income, age, education, and maternal skin color. Data collected from women in Rio Grande, Brazil, who gave birth in 2019, with follow-up from May 11 to July 20, 2020.

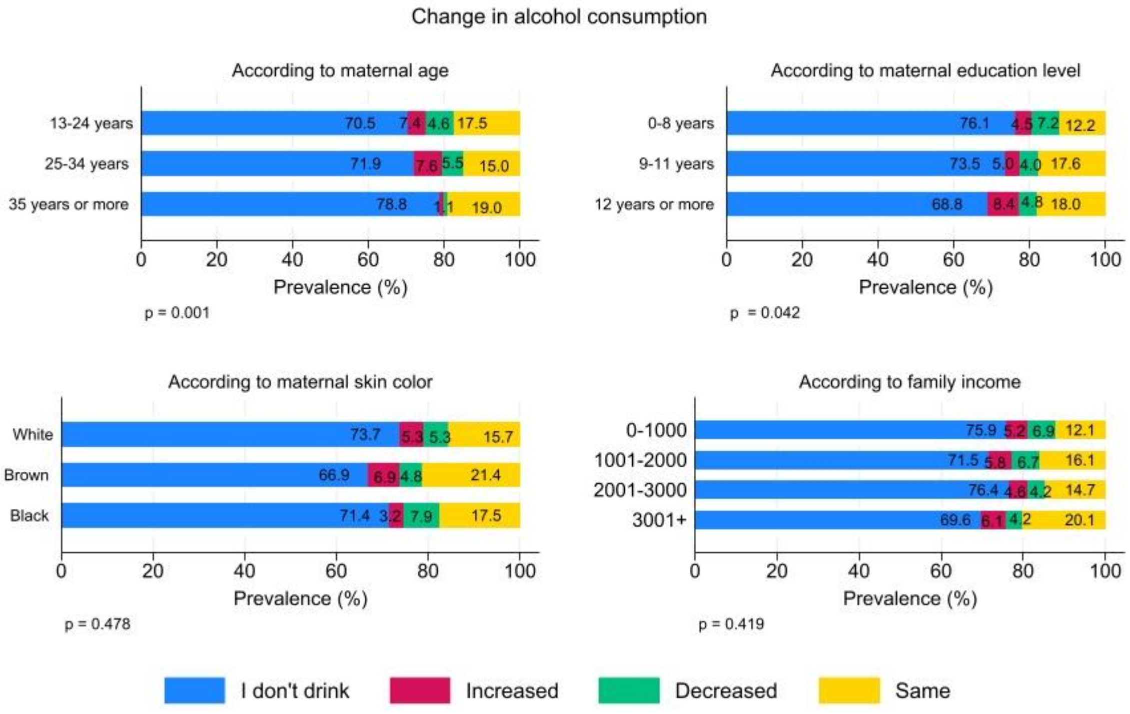

Supplementary Figure 5. Association between the change in the amount of food eaten and family income, age, education, and maternal skin color. Data collected from women in Rio Grande, Brazil, who gave birth in 2019, with follow-up from May 11 to July 20, 2020.

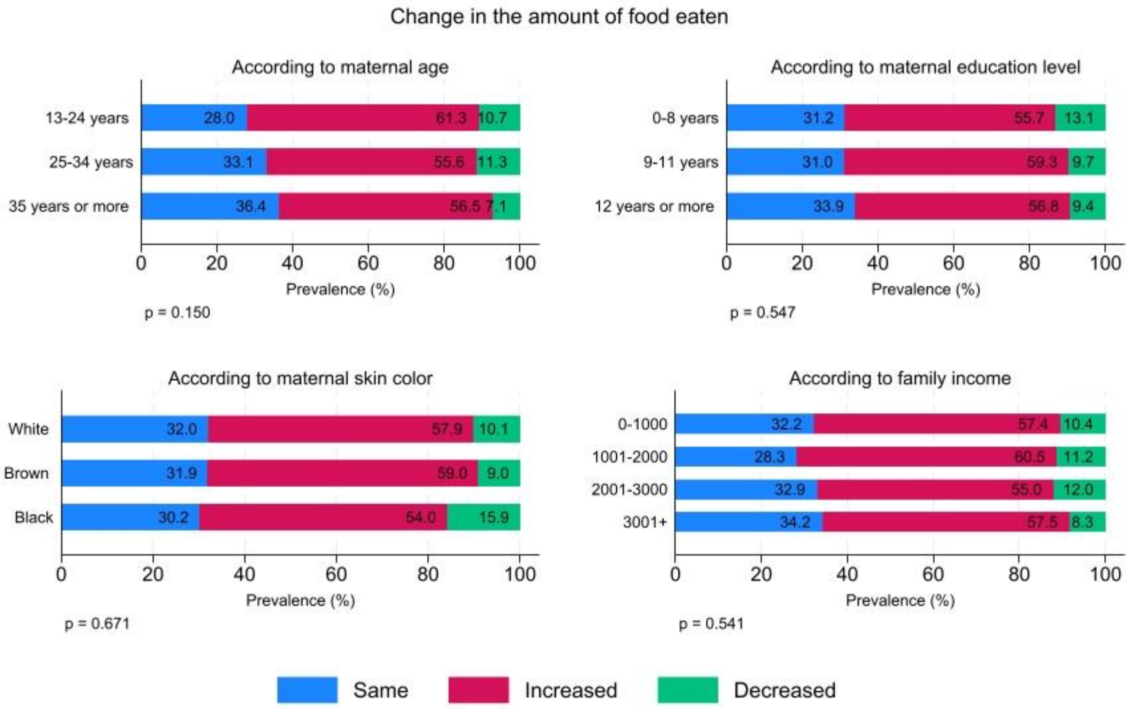

Supplement: Supplementary Table [file 1980-5497-rbepid-28-e250055-Suppl01.pdf]
